# Immune gene expression profiling of Proliferative Kidney Disease in rainbow trout *Oncorhynchus mykiss* reveals a dominance of anti-inflammatory, antibody and T helper cell-like activities

**DOI:** 10.1186/1297-9716-44-55

**Published:** 2013-07-16

**Authors:** Bartolomeo Gorgoglione, Tiehui Wang, Christopher J Secombes, Jason W Holland

**Affiliations:** 1 Scottish Fish Immunology Research Centre, School of Biological Sciences, University of Aberdeen, Tillydrone Avenue, Aberdeen AB24 2TZ, UK

## Abstract

The myxozoan *Tetracapsuloides bryosalmonae* is the causative agent of Proliferative Kidney Disease (PKD) targeting primarily the kidney of infected fish where it causes a chronic lymphoid immunopathology. Although known to be associated with suppression of some cellular aspects of innate immunity and a prominent lymphocytic hyperplasia, there remains a considerable knowledge gap in our understanding of the underlying immune mechanisms driving PKD pathogenesis. To provide further insights, the expression profiles of a panel of innate / inflammatory and adaptive immune molecules were examined in rainbow trout *Oncorhynchus mykiss* following a natural exposure to the parasite. Relative to controls, fish with early to advanced stages of kidney pathology exhibited up-regulation of the inflammatory cytokines interleukin (IL)-6 and IL-11, although remaining refractory towards genes indicative of macrophage activity. Antimicrobial peptides (AMPs) and anti-inflammatory markers, including cathelicidin (CATH) and IL-10 were markedly up-regulated during clinical disease. Up-regulation of adaptive immune molecules, including cell markers and antibody genes reflect the lymphocytic dominance of this disease and the likely importance of lymphocyte subsets in PKD pathogenesis. Up-regulation of T helper (T_H_) cell-like response genes and transcription factors implies that *T*. *bryosalmonae* may elicit a complex interplay between T_H_ cell subsets. This work, for the first time in the study of fish-myxozoan interactions, suggests that PKD pathogenesis is shaped by an anti-inflammatory phenotype, a profound B cell / antibody response and dysregulated T_H_ cell-like activities. A better understanding of the functional roles of fish immune cells and molecules in PKD pathogenesis may facilitate future development of control measures against this disease.

## Introduction

Proliferative Kidney Disease of salmonid fish is a slow progressive disease of major economic importance to aquaculture in the UK, Mainland Europe, and the USA [1,2]. Caused by the myxozoan parasite *Tetracapsuloides bryosalmonae*, PKD pathogenesis is a process closely linked to increasing water temperatures with climate change processes believed to impact on the latitude and altitude of the disease range [2]. Parasite spores, released from infected freshwater bryozoans, invade primarily via the gills and skin following the recognition of host nucleosides in mucous. Subsequently, parasites migrate through the vascular system to organs including the kidney and spleen with the former being the main target organ for further development [3,4]. Extrasporogonic proliferation in the kidney interstitium provokes a chronic immunopathology characterized by a lymphocytic hyperplasia, formation of granulomatous lesions, renal atrophy, and hyper secretion of immunoglobulins [1,2]. These stages are eventually eliminated in surviving fish and normal kidney function restored. Treatments with chemicals such as malachite green and fumigillin are effective against PKD, but these molecules are not licensable owing to their toxicity in humans [5,6]. Thus, in the absence of effective control measures immune therapy may be a way forward. However, this will necessitate an in-depth understanding of the immune mechanisms underlying the kidney immunopathology and protective immune responses to PKD. This is particularly poignant given that fish surviving PKD are immune to re-infection, providing the necessary impetus for vaccine development [1].

Previous studies examining host cellular responses during PKD have described a suppression of innate immune responses, including reduced phagocytic and respiratory burst activity of kidney phagocytes, reduced responsiveness to vaccination, and a dominance of immunoglobulin (Ig) M-negative lymphocytes that undergo *in situ* proliferation [7]. Until recently, the availability of fish immune genes for gene expression studies was mainly limited to innate immune processes such as pattern recognition, antimicrobial protein, and complement activities, and cytokines involved in pro-inflammatory responses (e.g. IL-1β and tumour necrosis factor (TNF)-α) [8]. With the advent of fish genome and EST databases, there has been a tremendous increase in the sequencing and characterization of fish immune genes, particularly those homologous to cellular markers and response genes associated with CD4^+^ and cytotoxic T cell activity in mammals, providing tantalizing insights into fish T cell biology [8,9]. Likewise, the discovery of the fish-specific mucosal immunoglobulin class, IgT, has uncovered a subset of IgT-specific B cells, known to respond specifically to the intestinal myxozoan parasite, *Ceratomyxa shasta* and to the protozoan *Ichthyophthirius multifiliis* following gill invasion [10-12]. The challenge now facing fish immunology is in the continued development of recombinant proteins and antibody markers, facilitating the functional characterization of fish immune responses and immune cell subsets involved in disease pathogenesis and protective responses. Given the current paucity of functional tools, expression profiling of fish immune genes represents a powerful means of providing insights into the immune mechanisms underlying disease pathogenesis and immune protective responses, pinpointing potential areas for future immunological intervention [13-15].

Our previous studies have indicated that both T_H1_ and T_H2_-like immune processes are involved in PKD pathogenesis, whilst lacking the classical signs of a pro-inflammatory response in fish exhibiting different stages of clinical disease [16-18]. Given the dominance of proliferating lymphocytes/antibody levels during PKD pathogenesis, the known suppression of phagocyte activity, the involvement of macrophages in the resolving stages of PKD and in response to other myxozoan parasites, this study was undertaken to further examine genes indicative of innate/inflammatory and adaptive immune responses. Based on the current availability and functional characterization of rainbow trout immune genes, we targeted genes that encompass innate/inflammatory/adaptive immune ligands, receptors, cell surface markers, antimicrobial peptides, and master transcription factors driving specific T_H_ cell responses in higher vertebrates. Thus, our study provides a balance between potential innate and adaptive immune mechanisms shaping PKD pathogenesis. In line with chronic immune pathologies mediated by mammalian extracellular parasites [19,20], our results suggest that PKD is characterized by a prevailing anti-inflammatory phenotype, over-expression of immunoglobulin isotypes, and a dysregulated T_H_-like response.

## Materials and methods

### Fish sampling and monitoring

Posterior kidney tissue samples were obtained from rainbow trout *Oncorhynchus mykiss* provided by a commercial trout farm in Southern England during a natural outbreak of PKD. The disease, at this site, is characterised by recurring annual epizootics with parasite detection in fish kidney smears occurring from late May to mid June depending on river temperature profiles, flow rates, and other environmental factors [2,21]. The bryozoan population(s) harbouring *T*. *bryosalmonae* at this site is not known. However, based on the dynamics of parasite release from known bryozoan populations, it is estimated that parasites are present in the water course feeding the farm from mid March [2,21]. Two groups of fish from the same egg source (*ca*. 50-100 g each) were sampled for this study. Firstly, a parasite-naïve uninfected group and secondly, a parasite-naïve group exposed to parasite-infected water from early April. Clinical signs of the disease were first seen in parasite-naïve exposed fish early June. Sampling of both groups was undertaken late July at a water temperature of 15-16 °C when naïve parasite-exposed fish exhibited kidney pathology ranging from early to advanced clinical stages (kidney swelling grades 1 to 3), as determined using the kidney swelling index system devised by Clifton-Hadley *et al*. [22]. All control fish had a kidney swelling grade of 0. In all fish sampled, approximately 100 mg of kidney tissue was removed immediately below the dorsal fin, the area of the kidney associated with the onset of clinical disease. Tissue samples were placed into 1 mL of RNA-later (Sigma, ST. Louis, USA), kept at 4 °C for 24 h and stored at −80 °C prior to RNA extraction and PCR analysis. The presence of *T*. *bryosalmonae* kidney stages in parasite-exposed fish was confirmed by histological examination of posterior kidney smears and by qPCR (see below). Checks for other pathogen infestations were conducted throughout the PKD season. With respect to fish used in this study, kidney swabs were streaked onto Tryptic Soy Agar (TSA) plates (Becton-Dickinson, Oxford, England) in order to check for the presence of common bacterial pathogens (e.g. *Aeromonas salmonicida*). Streaked plates were incubated for 48 h at 20-22 °C prior to examination for bacterial growth.

### RNA extraction and PCR analysis

Total RNA was extracted using TRI-reagent (Sigma) according to the manufacturer’s instructions. Purified RNA was quantified using a Nanodrop spectrophotometer (NanoDrop Technologies, Wilmington, USA) and reverse transcribed into cDNA (20 μg per sample) using Bioscript (Bioline, London, UK) in 30 μL reactions. cDNAs were diluted to 500 μL with TE buffer (pH 8.0) and stored at −20 °C. For qPCR analysis, cDNAs representing uninfected fish (*n* = 7) and fish exhibiting; early (grade 1; *n* = 6), moderate (grade 1–2; *n* = 9), and advanced (grade 2; *n* = 10 and grade 3; *n* = 10) stages of clinical disease were examined. Sixty primer sets were used encoding putative cellular markers and immune response genes, as well as primers for the reference gene elongation factor-1α (EF-1α). A full list of all primers and associated information is provided in Additional file 1. EF-1α has been repeatedly demonstrated to be highly consistent as a reference gene in the immune gene expression profiling of fish host-pathogen interactions [13-15,23]. The relative parasite prevalence in all tissue samples was assessed by qPCR using *T*. *bryosalmonae*-specific primers for the detection of the house-keeping genes; 18S rDNA [EMBL: U70623] and 60S ribosomal protein L18: RPL18 [EMBL: FR852769] [24]. 18S rDNA primers were modified relative to those in previous PKD studies [25] and were used to detect the parasite in gDNA samples. Although *T*. *bryosalmonae* RPL18 is highly homologous to the rainbow trout homologue (53% amino acid identity), *T*. *bryosalmonae*-specific RPL18 primers were designed within the open reading frame aided by the considerable difference in codon usage between host and parasite (typically, 45-55% GC and 25-40% GC respectively). Hence, detection of *T*. *bryosalmonae* RPL18 transcripts provides a sensitive measure of only viable parasites in each sample, whereas 18S rDNA detects both live and dead parasite material. With the exception of 18S rDNA, all primer pairs were designed and tested with a set of cDNA and DNA samples to ensure that products could only be amplified from cDNA and not from genomic DNA under the conditions used. Genomic DNA was extracted from the same TRI-reagent tissue homogenates used for RNA extraction, as described previously [13]. SYBR green (Invitrogen, Paisley, UK) based RT-qPCR using Immolase DNA Polymerase (Bioline) was performed using a Light Cycler® 480 SW 1.5 system (Roche, Mannheim, Germany) as described previously [26]. For parasite DNA detection, primer efficiency was determined using serial dilutions of reference (internal PCR control) DNA and used for quantification of the DNA concentration. To normalize the level of *T*. *bryosalmonae* 18S rDNA to the input genomic DNA, additional qPCR was undertaken using primers to the trout macrophage colony stimulating factor (MCSF) gene, as described previously [13]. For cDNA detection, the primer efficiency and concentration of each gene transcript was quantified using data generated from serially diluted reference DNA amplified in each PCR run, as described previously [13]. Since RPL18 mRNA detection is more sensitive than DNA detection and should more accurately reflect the viable parasite prevalence in individual fish, this was used for analysis of the immune gene expression data in addition to the kidney swelling grade assessment. To determine the RT-qPCR detection limit in terms of RPL18 transcript number, a pooled *T*. *bryosalmonae* positive sample was obtained from grade 2 cDNAs and serially diluted. A diluted RPL18 reference was included to enable relative quantification. The expression of trout immune genes was initially normalized to the expression of EF-1α and subsequently expressed as fold change relative to the expression level in parasite-naïve uninfected fish. Likewise, parasite RPL18 cDNA levels were normalized to that of trout EF-1α for each sample.

### Data analysis

Relative immune gene expression levels were anchored to the lowest value of each data set and Log2 transformed prior to statistical analysis, as described previously [27]. Correlations between *T*. *bryosalmonae* prevalence (RPL18 mRNA detection), kidney swelling grade, and immune gene expression were assessed by calculating the Pearson product–moment correlation coefficient (*r*) and considered significant at *P* ≤ 0.05 (2-tailed). The significance of the average fold change between uninfected and infected groups was analysed by one-way analysis of variance (ANOVA) and the LSD post hoc test for comparison of means with differences considered significant at *P* ≤ 0.05 (2-tailed). All statistical analyses were performed using SPSS® Statistics package v 20.0 (IBM Corporation, Somer, New York, USA) and graphically represented using GraphPad Prism version 5.04 (GraphPad Software Inc., La Jolla, USA).

## Results

### Pathogen detection

**Figure 1:**
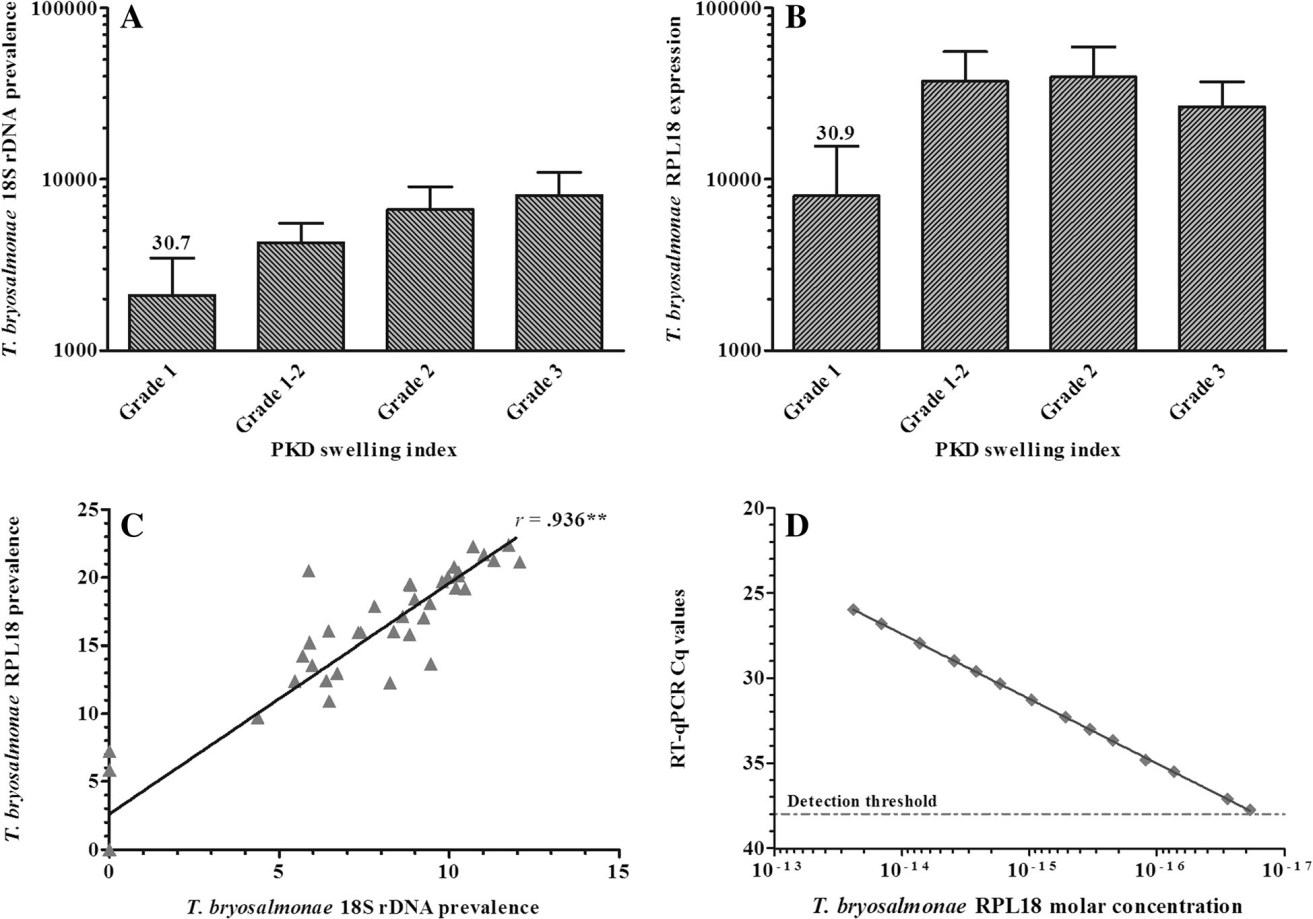
**Detection of *T*. *bryosalmonae *in rainbow trout kidney during clinical PKD. (A)** T. bryosalmonae 18S rDNA relative to trout MCSF (DNA detection); **(B)***T*. *bryosalmonae* RPL18 relative to trout EF-1α (mRNA detection). Average cCq value in grade 1 fish is provided above the bar; **(C)** Correlation between 18S rDNA and RPL18 parasite detection markers is shown (*r* = 0.94; *P* < 0.0001); **(D)** Linear relationship of RT-qPCR detection and molar concentration of *T*. *bryosalmonae* RPL18. A serial diluted RPL18 internal reference was included in the same PCR run to enable relative quantification.

Trout exposed to parasite-infected water were found to exhibit a range of clinical pathology ranging from grade 1 at low levels to grade 3 in cases of severe/advanced kidney pathology. 18S rDNA levels steadily increased in kidney tissue samples from grade 1 to grade 3 fish, reaching maximal levels in grade 3 fish whilst remaining undetectable in uninfected fish (Figure 1A). RPL18 expression, also undetectable in uninfected fish, plateaued at grades 1–2 and 2 decreasing by *ca*. 32% at grade 3 relative to grade 2 (Figure 1B). Nevertheless, parasite detection with 18S rDNA correlated highly with RPL18 (*r* = 0.936), with both significantly correlating with swelling grade (*r* = 0.755 and 0.740 respectively) (Figure 1C). A linear relationship was apparent between RT-qPCR detection and RPL18 molar concentration with a detection limit of 0.8 × 10^-17^ M (*ca*. 20 transcript copies in a 20 μL PCR reaction) (Figure 1D).

Importantly, agar plates prepared from kidney swabs from uninfected and *T*. *bryosalmonae* infected fish did not reveal the presence of bacterial pathogens, implying that the immune gene expression profiles observed in this study are influenced by the presence of *T*. *bryosalmonae*.

### Gene expression

A prerequisite in assessing the biological significance of altered gene expression profiles is to consider the expression levels indicated by average control Cq (cCq) values in uninfected fish. For example, cyclooxygenase (COX)-2B and inducible nitric oxide synthase (iNOS) transcripts in control fish are very rare (cCq: 32.7 and 33.3 respectively) with a *ca*. 4- to 5-fold increase unlikely to be biologically significant, whereas a 3-fold increase in the highly abundant transforming growth factor (TGF)-β1a transcript (cCq: 22.9) is likely to be of greater biological significance. Therefore, in addition to fold changes in gene expression in grade 1 to 3 fish, the average cCq values for the genes studied (i.e. in grade 0 fish) are presented in Table 1 and in Additional file 2.

**Table 1:** **Summary of immune gene expression in *T***. ***bryosalmonae *infected rainbow trout kidney tissue**

**Gene**	**Grade 0 average cCq**	**Kidney swelling grade**				
		**Grade 1**	**Grade 1-2**	**Grade 2**	**Grade 3**	**Pearson correlation**
***T. bryosalmonae *18S rDNA**	*Not detectable*	2105.69**	4266.94**	6652.34**	8034.26**	.755**
***T. bryosalmonae *RPL18**		8013.47**	37233.66**	39385.81**	26654.46**	.740**
**IL-1β-1**	27.16	3.71*	2.24	2.10	2.31	-.116
**TNF-α1**	24.52	1.12	0.95	0.94	0.84	-.155
**TNF-α2**	26.60	0.97	2.19*	1.52	1.64	.106
**IL-6**	34.12	7.44**	31.53**	23.59**	15.38**	.510**
**IL-11**	29.62	31.74**	25.04**	14.13**	11.54**	.399**
**M17**	26.05	5.10**	6.28**	5.48**	2.60	.179
**COX-2A**	24.69	2.15	1.68	1.75	1.07	-.161
**COX-2B**	32.73	3.59	3.94*	3.10*	2.03	.164
**MCSF-1**	22.92	1.18	0.77	0.49*	0.23**	-.658**
**MCSF-2**	21.78	1.81	3.82*	2.77	1.41	-.076
**MCSF-R1**	27.65	1.05	2.48	1.01	0.57*	-.375*
**MCSF-R2**	21.77	2.15	2.70	2.65	1.22	-.001
**iNOS**	33.31	2.34	5.54*	2.43	1.28	-.035
**Arginase-1**	15.20	1.05	0.50*	0.45*	0.23**	-.612**
**Cathelicidin-1**	28.63	52.60**	136.21**	72.80**	57.81**	.530**
**Cathelicidin-2**	29.58	9.00**	56.40**	94.90**	19.10**	.547**
**Hepcidin-1**	25.76	5.36*	10.85*	22.37**	6.42**	.360*
**LEAP-2A**	34.31	10.92**	12.87**	7.76**	6.84**	.281
**CD8α**	25.31	5.37**	4.01**	5.46**	3.91*	.238
**CD8β**	21.41	2.99*	3.05*	3.34*	2.33	.128
**IL-2Rβ**	24.63	3.79**	5.19**	1.28	0.93	-.168
**IgD H-secretory**	21.39	0.98	1.02	0.89	0.92	-.095
**IgM H-secretory**	14.14	5.39*	3.38	8.80**	4.80**	.399**
**IgT H-secretory**	21.31	8.19*	24.68**	51.03**	68.62**	.732**
**CD4**	22.47	3.66**	4.28**	4.72**	3.56*	.298
**T-bet**	24.94	7.00**	6.86**	7.06**	2.80**	.203
**IL-2**	28.68	3.68*	4.16**	2.16	2.54	.048
**IFNγ**	29.25	5.06**	15.40**	10.03**	8.49**	.500**
**GATA3**	25.00	4.64**	5.77**	3.63*	2.33	.115
**IL-4/13A**	25.77	1.67	2.71	1.70	0.80	-.124
**IL-21**	33.78	20.28**	42.62**	31.58**	11.91**	.506**
**IL-22**	33.65	10.03*	6.67**	5.49*	4.48	.159
**IL-17A/F2a**	28.27	10.84**	11.23**	2.64	3.24	.061
**IL-17C1**	27.92	6.56**	2.74*	1.43	0.84	-.259
**IL-17C2**	30.90	2.08	1.36	1.06	0.70	-.349*
**IL-17D**	31.72	4.95**	5.73**	3.78**	3.56*	.259
**FOXP3A**	26.19	2.96	5.15*	4.75	2.52	.042
**FOXP3B**	27.03	4.02**	3.87**	3.13**	2.11	.109
**TGF-β1a**	22.87	3.34*	3.00**	2.02	1.59	-.015
**nIL-1F**	24.76	3.14*	3.32**	2.81*	1.97	.136
**IL-10A**	26.26	14.68**	18.21**	25.24**	9.51**	.414**
**SOCS-3**	22.74	3.44*	8.38**	10.24**	5.90**	.472**

The average base-line Cq (cCq) in control (grade 0) fish is provided for each gene studied with the exception of parasite genes, which were undetectable in control fish. Immune gene expression at each stage of clinical PKD (grade 1 to 3) was initially normalized to trout EF-1α and subsequently expressed as fold change relative to expression levels in control fish. Pearson correlation *r* coefficients are given relative to kidney swelling grade. Significant differences (2-tailed) between control and infection groups are shown in bold. * *P* < 0.05, ** *P* < 0.01.

**Figure 2:**
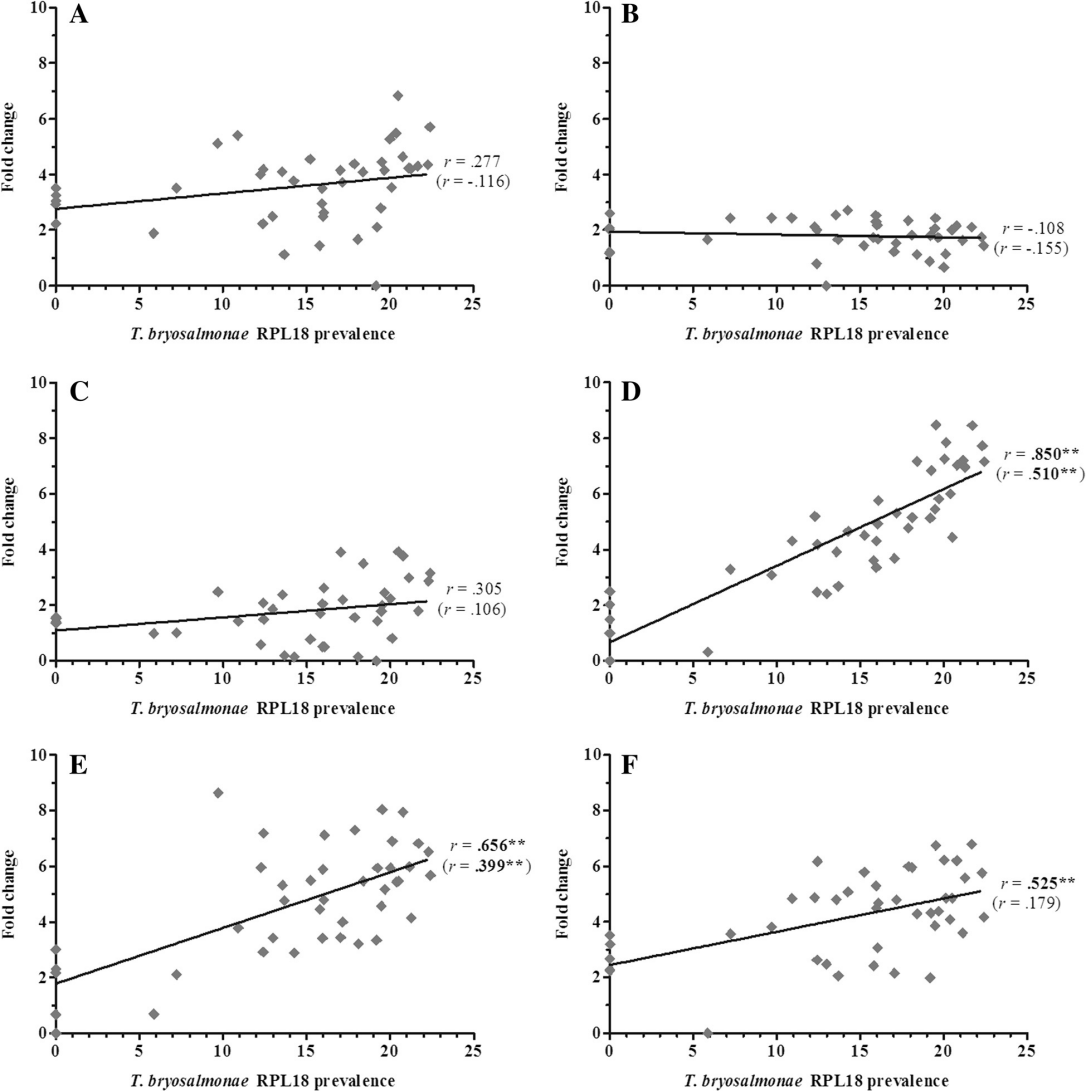
**Correlation of *T. bryosalmonae *prevalence and inflammatory genes in rainbow trout kidney. (A)** IL-1ß-1; **(B)** TNF-α1; **(C)** TNF-α2; **(D)** IL-6; **(E)** IL-11; **(F)** M17. Pearson correlation *r* coefficients are given relative to *T*. *bryosalmonae* RPL18 and kidney swelling (in parentheses). Significant correlations are shown in bold. *P < 0.05; **P < 0.01 (2-tailed).

**Figure 3:**
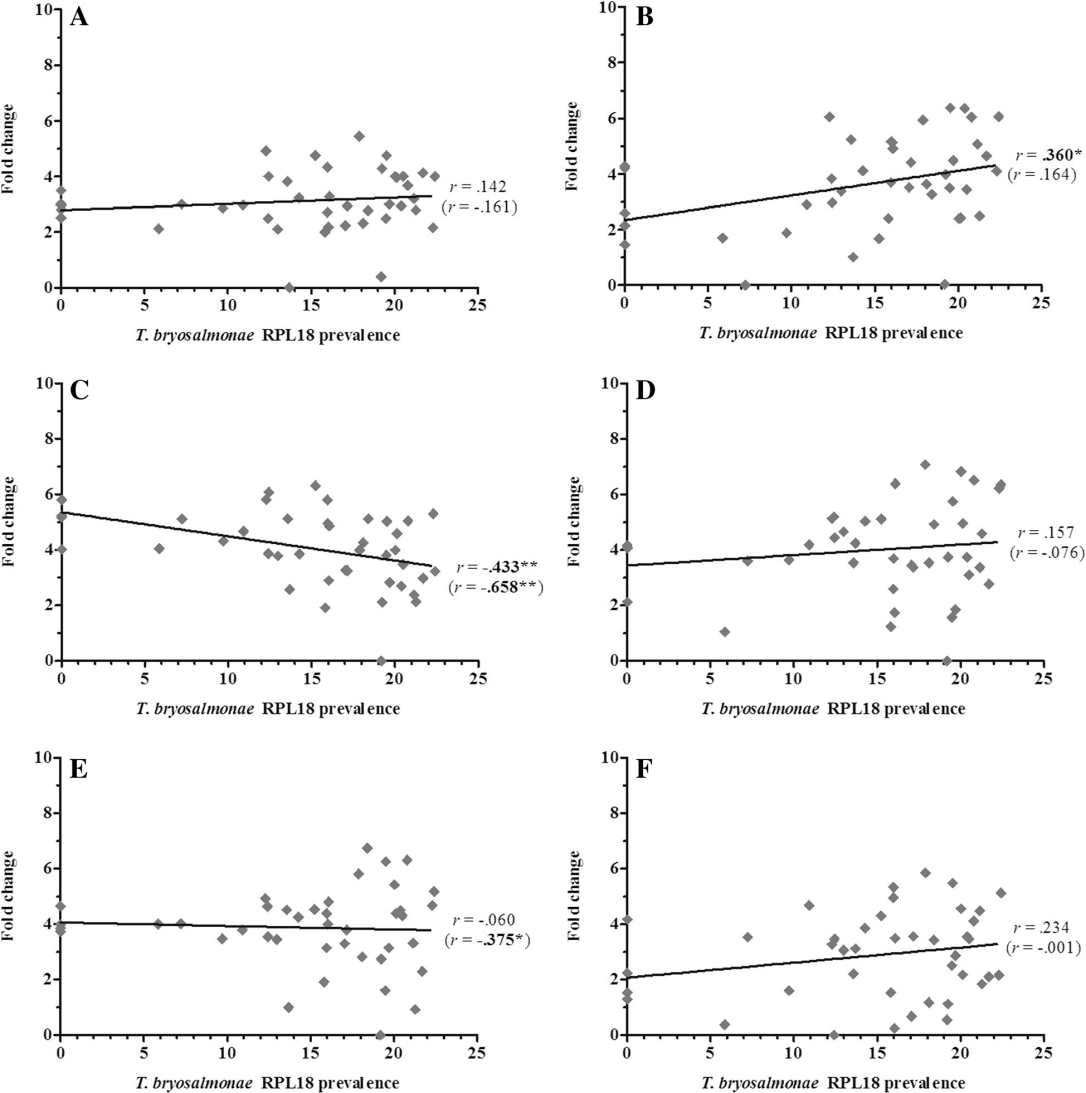
**Correlation of *T. bryosalmonae *prevalence and macrophage marker/response genes in rainbow trout kidney. (A)** COX-2A; **(B)** COX-2B; **(C)** MCSF-1; **(D)** MCSF-2; **(E)** MCSF-R1; **(F)** MCSF-R2. Pearson correlation *r* coefficients are given relative to *T*. *bryosalmonae* RPL18 and kidney swelling (in parentheses). Significant correlations are shown in bold. *P < 0.05; **P < 0.01 (2-tailed).

**Figure 4:**
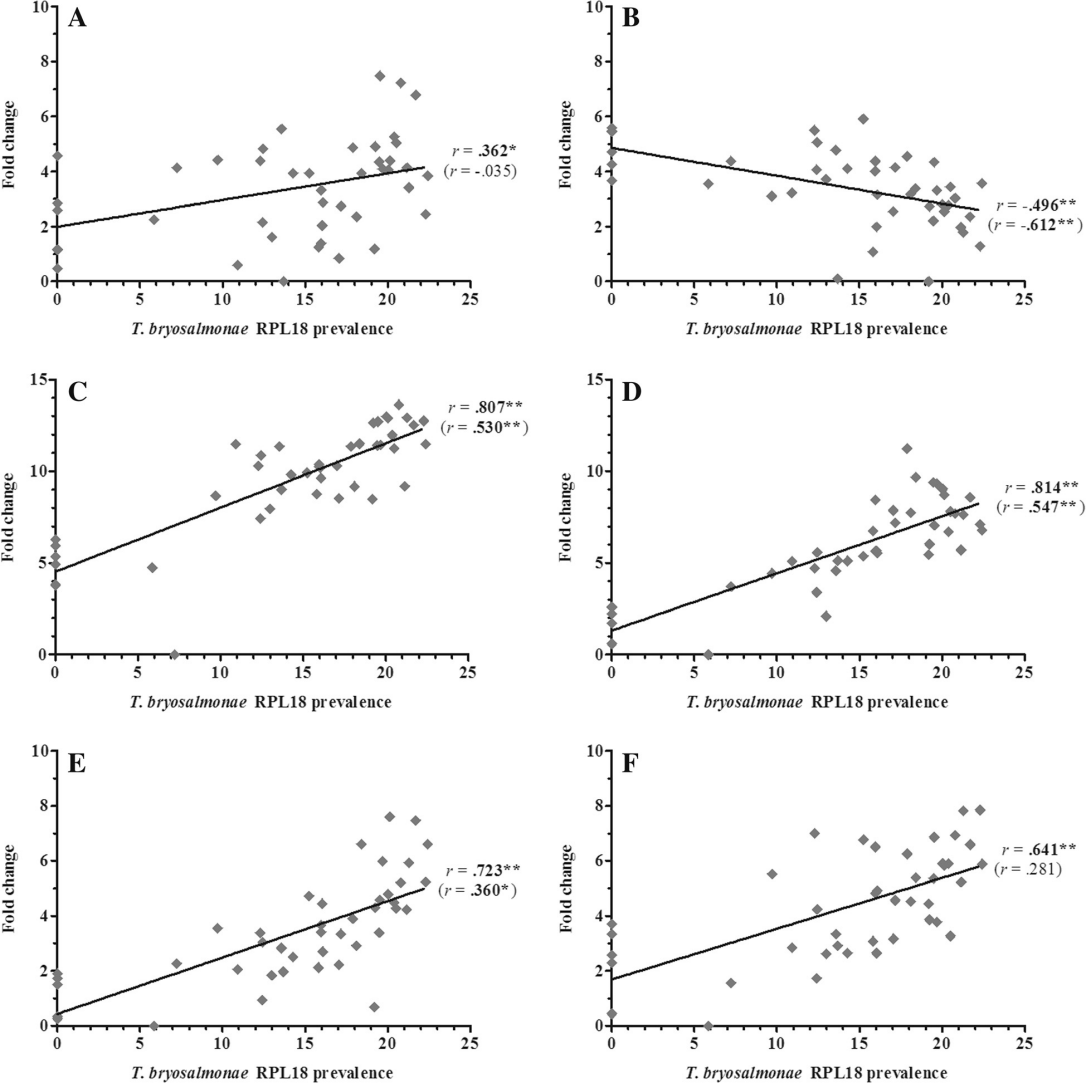
**Correlation of *T. bryosalmonae *prevalence and antimicrobial genes in rainbow trout kidney. (A)** iNOS; **(B)** Arginase-1; **(C)** Cathelicidin-1; **(D)** Cathelicidin-2; **(E)** Hepcidin-1; **(F)** LEAP-2A. Pearson correlation *r* coefficients are given relative to *T*. *bryosalmonae* RPL18 and kidney swelling (in parentheses). Significant correlations are shown in bold. *P < 0.05; **P < 0.01 (2-tailed).

**Figure 5:**
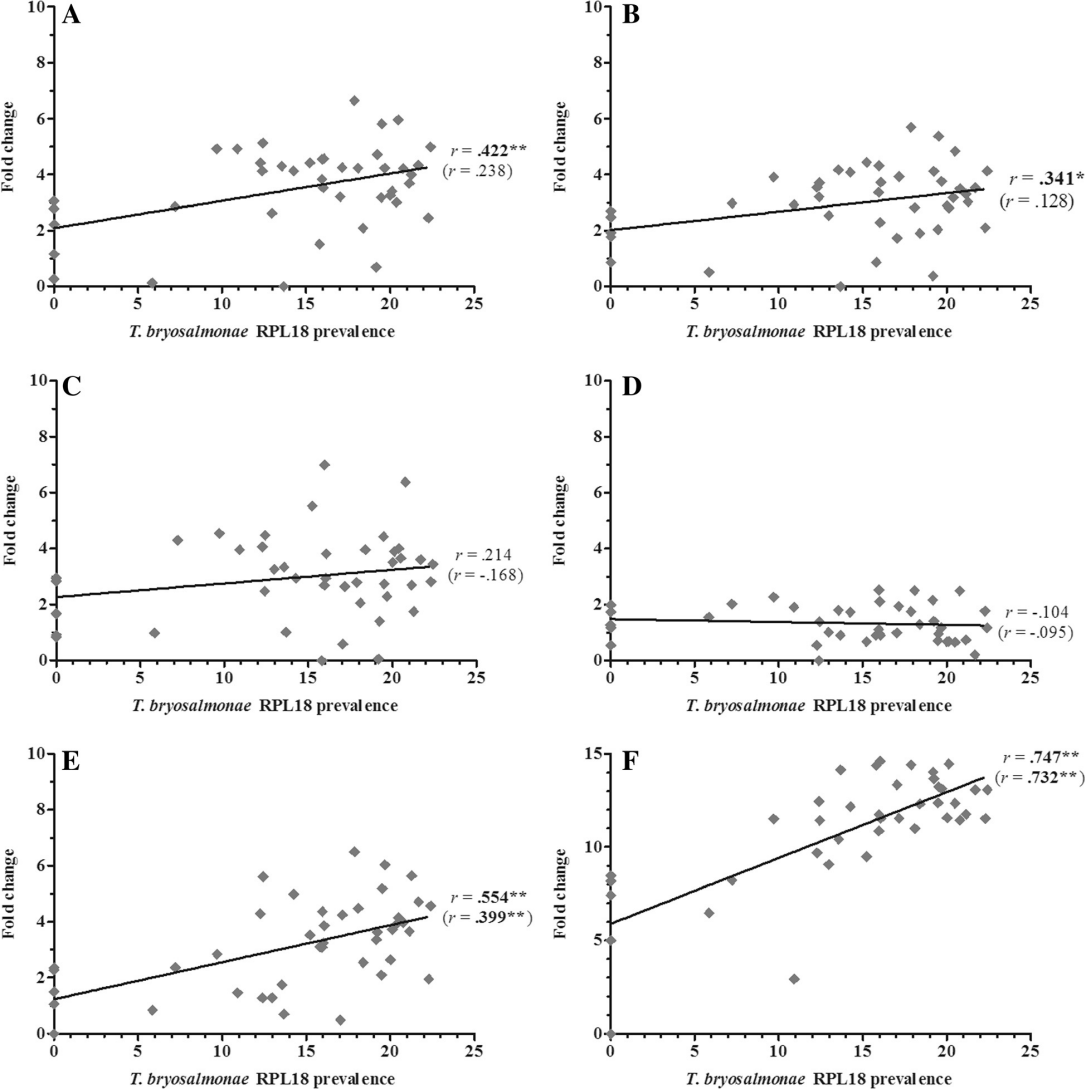
**Correlation of *T*. *bryosalmonae *prevalence and cell/antibody marker genes in rainbow trout kidney. (A)** CD8α; **(B)** CD8ß; **(C)** IL-2Rß; **(D)** secretory IgD; **(E)** secretory IgM; **(F)** secretory IgT. Pearson correlation *r* coefficients are given relative to *T*. *bryosalmonae* RPL18 and kidney swelling (in parentheses). Significant correlations are shown in bold. *P < 0.05; **P < 0.01 (2-tailed).

**Figure 6:**
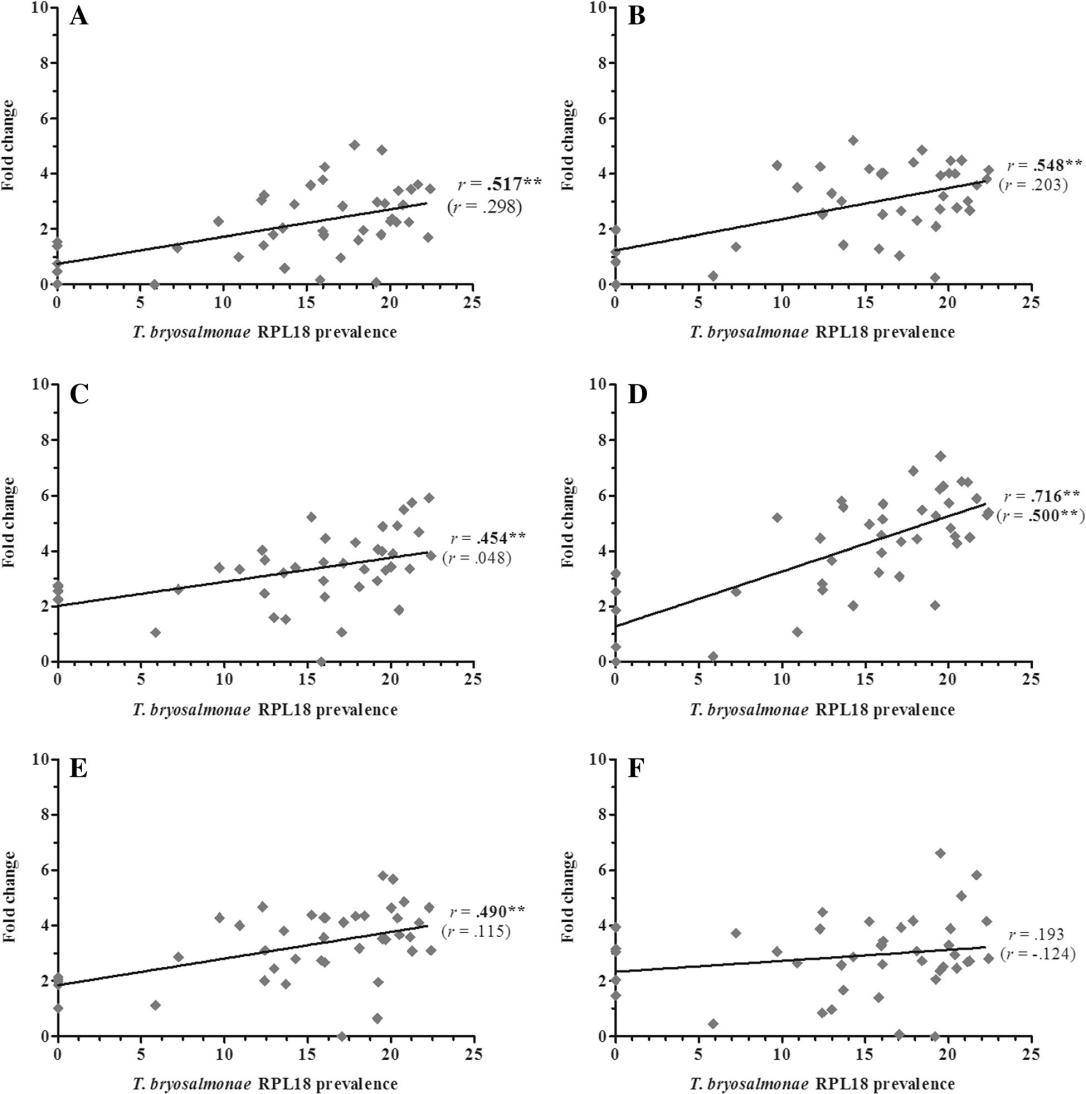
**Correlation of *T. bryosalmonae *prevalence and T**_**H **_**marker genes in rainbow trout kidney. (A)** CD4; **(B)** Tbet; **(C)** IL-2; **(D)** IFNγ; **(E)** GATA3; **(F)** IL-4/13A. Pearson correlation *r* coefficients are given relative to *T*. *bryosalmonae* RPL18 and kidney swelling (in parentheses). Significant correlations are shown in bold. *P < 0.05; **P < 0.01 (2-tailed).

**Figure 7:**
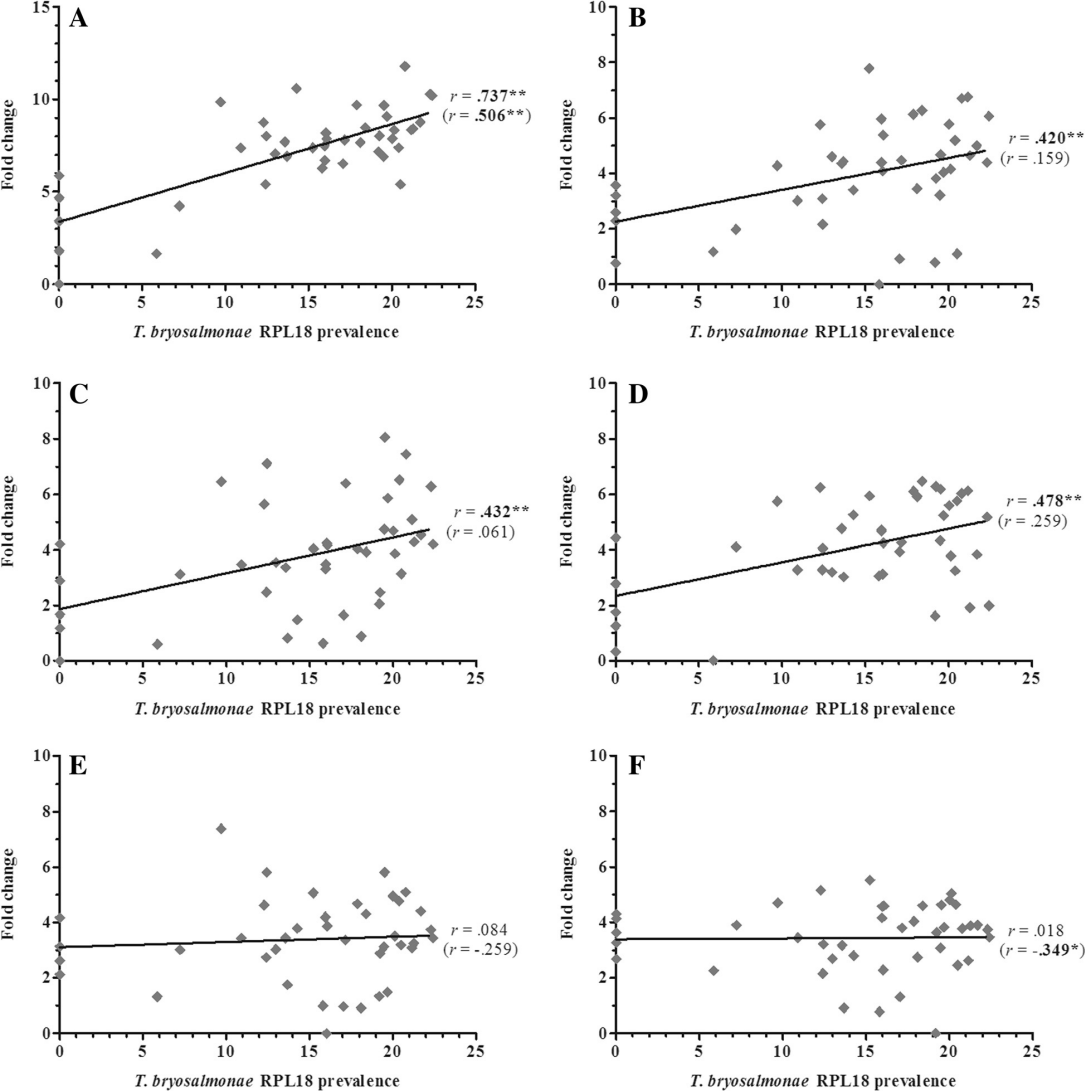
**Correlation of *T. bryosalmonae *prevalence and T**_**H**_**-17 marker genes in rainbow trout kidney. (A)** IL-21; **(B)** IL-22; **(C)** IL-17 A/F2a; **(D)** IL-17D; **(E)** IL-17C-1; **(F)** IL-17C-2. Pearson correlation *r* coefficients are given relative to *T*. *bryosalmonae* RPL18 and kidney swelling (in parentheses). Significant correlations are shown in bold. *P < 0.05; **P < 0.01 (2-tailed).

**Figure 8:**
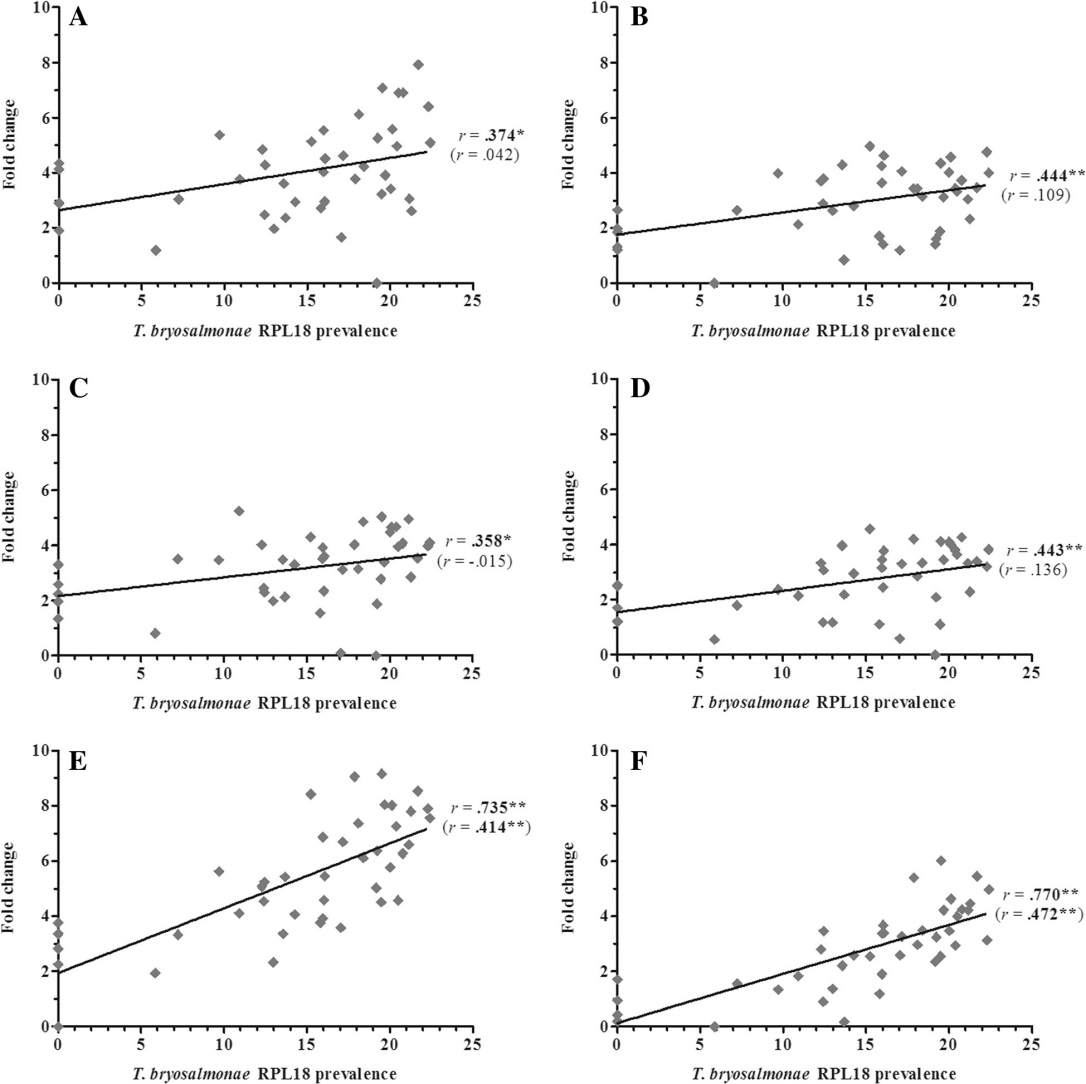
**Correlation of *T. bryosalmonae *prevalence and T**-**reg/anti**-**inflammatory genes in rainbow trout kidney. (A)** FOXP3A; **(B)** FOXP3B; **(C)** TGF-ß1a; **(D)** nIL-1F; **(E)** IL-10A; **(F)** SOCS-3. Pearson correlation *r* coefficients are given relative to *T*. *bryosalmonae* RPL18 and kidney swelling (in parentheses). Significant correlations are shown in bold. *P < 0.05; **P < 0.01 (2-tailed).

Overall, 36 gene transcripts correlated positively with parasite RPL18 transcript levels, with 9 genes (IL-6, IL-10A, IL-21, IgT (secretory), interferon (IFN) γ, suppressor of cytokine signalling (SOCS)-3, CATH-1, CATH-2 & hepcidin-1) strongly correlated (*r* ≥ 0.7) (Figures 2, 3, 4, 5, 6, 7 and 8. See Additional file 2 for IL-18, IgT (membrane), type I IFN-A, IL-10B, SOCS-1, and IL-15 data). Sixteen genes correlated positively with both parasite RPL18 and kidney swelling, including all genes strongly correlating with RPL18, together with IL-10B, IL-11, MCSF-1, arginase-1, IgM (secretory), IgT (membrane) and SOCS-1. Conversely, four genes correlated with the kidney swelling grade and not parasite RPL18, namely; MCSF-R1, RAR-related orphan receptor (ROR)γ, IL-17C-2 and SOCS-2 (Figures 3 and 7. See Additional file 2 for RORγ and SOCS-2 data). In each case a weak negative correlation was evident. Below, we focus on those genes correlating with parasite RPL18 expression, 55.6% (20 genes) of which would not have been detected by examining potential correlations between immune gene expression and kidney swelling grade alone.

Expression of genes encoding the classical pro-inflammatory cytokines, IL-1β and TNF-α did not correlate with parasite prevalence or kidney swelling grade regardless of the paralogues studied (i.e. IL-1β-1 or IL-1β-3; TNF-α1 or TNF-α2). (Figure 2. See Additional file 2 for IL-1β-3 data). In contrast, the inflammatory cytokines IL-6, IL-11, IL-18 and M17 all correlated with parasite prevalence, with IL-6 markedly so (*r* = 0.85). IL-6 and IL-11 gene expression also correlated with kidney swelling grade (Figure 2). Another proinflammatory mediator, COX-2, also showed a significant correlation with parasite prevalence (Figure 3), but only with respect to COX-2B. Both MCSF-1 and arginase-1 exhibited a significant negative correlation with both parasite prevalence and kidney swelling grade (Figures 3 and 4). The expression of both isoforms of the MCSF receptor (MCSF-R1/2) did not correlate with parasite prevalence and only weakly correlated with kidney swelling grade in the case of MCSF-R1 (Figure 3). Similarly, the expression of a marker of mature antigen presenting cells (CD83) did not correlate with either infection read-out. Gene expression of the antimicrobial peptides examined correlated with parasite prevalence, strongly in the case of CATH-1/2 and hepcidin; furthermore, these genes also correlated significantly with kidney swelling grade (Figure 4).

To examine the potential contribution of the adaptive immune system to PKD pathogenesis, the expression of genes homologous to mammalian B and T cell markers and signature molecules of T_H_ responses were investigated. Expression of secretory forms of IgM and IgT correlated positively with parasite prevalence and kidney swelling grade, with IgT correlating strongly (*r* = 0.747 and 0.732 respectively) (Figure 5). The membrane form of IgT also exhibited a positive, albeit weak, correlation with both infection read-outs, whilst membrane IgM was refractory. In contrast, membrane and secretory forms of IgD were refractory to both infection read-outs with no PKD-mediated gene modulation detected (Figure 5 Additional file 2). Expression of the T cell markers CD4 and CD8 (subunits α and β) exhibited a positive correlation with parasite prevalence but not with kidney swelling grade. Strikingly, the IL-2 receptor chain, IL-2Rβ although not correlating with either infection read-out was up-regulated at early clinical stages, dropping sharply at grades 2 and 3, as shown in Figures 5 and 6, and Additional file 2.

In depth analysis of genes encoding T_H1_ signature molecules revealed a putative T_H1_-like component to PKD pathogenesis. The master T_H1_ T cell-specific T-box transcription factor, T-bet, was positively correlated with parasite prevalence (Figure 6). The expression of IFN-γ and IL-2 correlated with parasite prevalence, with IFN-γ also correlating with kidney swelling grade (Figure 6). As shown in Additional file 2, another important growth factor for T cells, namely IL-15, was upregulated during early clinical stages, correlating positively with parasite prevalence. The master T_H2_ trans-acting T cell-specific transcription factor, GATA3, exhibited a similar expression profile to T-bet (Figure 6). However, expression of the trout IL-4/13 cytokine family member, IL-4/13A, was not affected by infection (Figure 6). In the context of T_H17_ cell-derived cytokines, we have analysed the transcriptional profiles of the four IL-17 family members found in trout, namely, IL-17A/F2a, IL-17C-1/C-2, and IL-17D (Figure 7). IL-17A/F2a, IL-17C-1, and IL-17D exhibited only weak or no correlation with parasite prevalence and no correlation with kidney swelling (Figure 7). Strikingly, however, in a similar manner to IL-2Rβ, IL-17A/F2a and IL-17C-1 were both upregulated at early clinical stages, dropping sharply at more advanced stages (Table 1). Expression of the trout homologue of the T_H17_ master transcription factor, RORγ did not correlate with either infection read-out. In contrast, the T_H17_ cytokine, IL-21 strongly correlated with parasite prevalence (*r* =0.737) and significantly, although to a lesser extent, with kidney swelling grade whereas, IL-22 correlated only with parasite prevalence (Figure 7).

A range of anti-inflammatory immune genes was also studied, including IL-10 paralogues, TGF-β1a, the trout IL-1β antagonist nIL-1F, paralogues of the master T_reg_ transcription factor, forkhead box (FOX) P3, and several members of the SOCS family (Figure 8. See Additional file 2 for SOCS-1, -2, -5b, -7 data). The expression of both IL-10 paralogues strongly correlated with parasite prevalence (IL-10A: *r* = 0.735 and IL-10B: *r* = 0.679) and weakly with kidney swelling grade, whilst the expression of TGF-β1a, nIL-1F, and FOXP3A/B exhibited weak or no correlation with both infection read-outs (Figure 8. See Additional file 2 for IL-10B data). Of the SOCS molecules examined, SOCS-1 and SOCS-3 correlated significantly with parasite prevalence and kidney swelling grade (Figure 8. See Additional file 2 for SOCS-1 data). Lastly, four other genes of relevance to inflammatory processes were studied, namely CD9, type I IFN, vascular endothelial growth factor (VEGF), and ciliary neurotrophic factor (CNTF). As shown in Additional file 2, type I IFN exhibited a weak correlation with parasite prevalence, but no correlation with kidney swelling grade. All other genes were refractory towards both infection read-outs.

## Discussion

Until recently, the paucity of available fish immune genes, particularly those indicative of T_H_ cell-like activities, has hampered gene expression studies investigating fish host-pathogen interactions. Here, for the first time in the study of host-myxozoan interactions, we present gene expression data providing insights into the underlying innate and adaptive immune mechanisms shaping the pathogenesis of PKD in trout. Such knowledge may pinpoint immune molecules and pathways that could be targeted for immunological intervention.

In general, immune gene expression changes were more strongly correlated with parasite prevalence than kidney swelling. With respect to parasite prevalence, the apparent reduction in viable parasites at swelling grade 3 from the parasite RPL18 data relative to 18S rDNA could signify the activation of mechanisms leading to pathogen clearance and recovery. Differences between parasite prevalence and kidney swelling grade are likely to be due to the fact that, for each fish, parasite prevalence and immune gene expression data were collated from the same tissue sample, whereas kidney swelling data is based on a qualitative holistic assessment of disease progression. The modulation of nine genes correlated strongly with parasite prevalence with IL-6, CATH-1, and CATH-2 exhibiting *r* values > 0.8. Recent studies describing functional associations between immune gene expression and parasite prevalence in wild host populations suggest that they may reflect host-parasite co-evolutionary processes [28]. Similarly, trout immune genes strongly correlating with *T*. *bryosalmonae* prevalence in the current study may exemplify host genes under selection.

### Innate immunity / inflammatory mediators

The cytokines, IL-1β and TNF-α, are crucial mediators of pro-inflammatory responses and in the activation of B and T cells [29]. Functionally assessed to have pro-inflammatory activity in fish, IL-1β and TNF-α are often co-expressed with other macrophage-derived inflammatory mediators such as IL-8, COX-2, and iNOS in parasitic and bacterial infections [13-15,30,31]. Our previous studies revealed no or a very modest impact of PKD on the gene expression of pro-inflammatory mediators, which has been corroborated by the current study [16]. Similarly, other myxozoan fish parasites appear to elicit either weak transient up-regulation or, indeed, down-regulation of genes encoding pro-inflammatory mediators as part of a global down-regulation of innate and acute phase response genes [32,33].

In the current study, we extended the repertoire of inflammatory mediators to include IL-6, IL-10, IL-11, TGFβ1, and the IL-1β antagonist, nIL-1F. Intriguingly, IL-6 and IL-11 transcripts were up-regulated over 30-fold correlating positively with both infection readouts. Unlike IL-1β and TNF-α, IL-6 exhibits both pro- and anti-inflammatory activities in the regulation of macrophages and lymphocytes [34]. Trout studies have also attributed pro- and anti-inflammatory activities to fish IL-6 in stimulating macrophage proliferation and induction of cytokine inhibitors (SOCS-1/-3), antimicrobial peptides (CATH-2, hepcidin, & liver-expressed antimicrobial peptide-2 isoform A; LEAP-2A), whilst down-regulating TNF-α and IL-1β [35]. Furthermore, PKD elicited transcriptional up-regulation of the fish-specific IL-6 family member, M17 which correlated positively with parasite prevalence [36]. We previously described PKD-associated up-regulation of SOCS 1/3 genes [18]. Thus, marked up-regulation of CATH-1/-2 and LEAP-2A along with the unresponsiveness of TNF-α and IL-1β in the current study could be, in part, due to the activity of trout IL-6 family members. Fish IL-10 has been shown to be anti-inflammatory, suppressing pro-inflammatory cytokine expression, phagocytosis, and respiratory burst activity [37]. Considering the dominance of IL-10 gene expression during PKD pathogenesis, this cytokine could be an important player in the PKD-mediated suppression of phagocytic activity [7,37]. Interleukin 11, also markedly influenced during PKD, is considered to be a potent anti-inflammatory cytokine in mammals [38]. Although not functionally characterised in fish to date, IL-11 could, nevertheless, also play an important role in the suppression of pro-inflammatory mediators and innate immunity during PKD pathogenesis.

The apparent lack of a pro-inflammatory response to *T*, *bryosalmonae* at the transcriptional level is particularly intriguing given that fish bacterial and other parasitic infections exhibit, to some extent, concomitant up-regulation of pro-inflammatory (TNF-α, IL-1β, IL-8) and anti-inflammatory (IL-6, IL-10, IL-11, nIL-1F) cytokines [13-15]. This disparity could be attributed to the characteristic lymphocytic dominance of PKD pathogenesis relative to other fish diseases, although macrophages are as prominent as lymphocytes in granulomatous lesions that develop during the resolving stages of PKD [1]. The putative lack of a macrophage-driven pro-inflammatory response to *T*. *bryosalmonae* is further reinforced by the refractoriness or, indeed, down-regulation of other macrophage marker genes such as arginase-1 and MCSF ligand/receptor paralogues [39].

A surprising finding in this study was the strong correlation between parasite prevalence and the prominent transcriptional up-regulation of the antimicrobial peptides, CATH-1, CATH-2, and hepcidin-1. Antimicrobial peptide expression is known to be influenced by parasite infection in both vertebrate and invertebrate host-parasite interactions. Salmonid CATH-1 and/or CATH-2 genes are markedly up-regulated by both bacterial and parasitic infection [14,23,35]. CATH-1/-2 peptides have been ascribed direct antibacterial and indirect cytokine modulating activities in fish, whilst a CATH-2 peptide, was able to slow oomycete sporulation [14,40]. These studies highlight the potential for fish cathelicidins to protect against *T*. *bryosalmonae* directly or indirectly through the modulation of cytokine activity. As in fish, mammalian cathelicidins are influenced by bacteria and parasites mediating both pro- and anti-inflammatory responses. Although considered beneficial, they may also contribute to infection-mediated immunopathology [41]. Thus, cathelicidins may add to the apparent anti-inflammatory nature of PKD and to the associated pathogenesis. Hepcidin, an iron regulator that reduces the availability of iron for pathogen growth, is up-regulated by bacterial and parasitic infection in salmonids [14,23,42]. Although not functionally characterized in fish, an important role for this molecule in the control of mammalian parasitic infections has been recently demonstrated [43]. As with cathelicidins, fish hepcidin is likely to have a broad spectrum of activity against fish pathogens.

### Adaptive immunity

Suppression of innate immune/phagocytic activity and *in situ* proliferation of lymphocytes are two of the most significant cellular aspects of clinical PKD [7]. An over-shadowing anti-inflammatory phenotype may account for the aberrant lymphocytic response through suppression of phagocyte activity. Hyperimmunoglobulaemia is also a major characteristic of PKD with immunoglobulins also playing a major role in other myxozoan infections [1,10]. Here we report the presence of very high levels of transcripts encoding the pathogen-responsive immunoglobulin isotypes, IgM and IgT. All Ig primer sets were designed in the heavy chain constant region. We, therefore, acknowledge that we were unable to distinguish between viable and sterile Ig transcripts. However, we know from recent trout studies that the abundance of viable Ig transcripts during infection processes is *ca*. 70% [44]. Intriguingly, the membrane form of each Ig subtype was not or very weakly influenced whereas the secretory form was markedly up-regulated, which agrees with the IgM negative nature of the proliferating lymphocytic population in the kidneys of *T*. *bryosalmonae*-infected fish [7]. Our data corroborates with previous reports of hyperimmunoglobulinaemia in that Ig secretion appears to be a major feature of PKD pathogenesis [1]. The absence of up-regulated membrane Ig transcripts may suggest that B cells are not undergoing proliferation during the course of PKD and that the IgM negative proliferating lymphocyte population associated with PKD pathogenesis could be attributed to the proliferation of T cell subsets. This would, however, need to be further substantiated using specific antibodies to trout IgT and T cell markers when available. In support of this premise, we observed significant transcriptional up-regulation of CD4 and CD8 suggesting a potential role for T_H_ and T_C_-like responses in PKD pathogenesis. Aberrant T cell activity, could to some extent account for the over-expression of antibody isotypes as seen in mammalian parasite-mediated chronic immunopathologies [20].

To investigate T cell-like activity in PKD, the expression of trout genes homologous to mammalian molecules defining T_H1_, T_H2_, T_H17_, and T_reg_-like responses were studied [9]. Mammalian pathogen-mediated chronic immunopathologies have been linked to dysregulated T_H_ responses involving a complex interplay between stimulatory and suppressive immune signals leading to pathology rather than parasite clearance [19,45]. IL-17A is one of the main drivers of parasite-mediated pathology, particularly in the formation of granulomatous tissue, a process regulated by IL-10, TGF-β and T_reg_ cell activity. Reducing IL-17A in this context enhances protective mechanisms by reducing pathology/parasite prevalence and enhancing parasite-specific antibodies [19]. Furthermore, T_reg_ responses are thought to be responsible for T cell anergy under such conditions resulting in reduced responsiveness to bystander vaccines [20]. PKD also entails the formation of granulomatous lesions and reduced responsiveness to by-stander vaccines [7]. Thus, fish T_H_-like activities may play an integral role in shaping this disease. In line with previous fish studies, T-bet, the master T_H1_ transcription factor and the T_H1_ signature cytokines, IFN-γ and IL-2 (including receptor) were up-regulated during PKD [13,15,17]. Both cytokines are known to drive T_H1_-like responses in fish, which implies that in the context of adaptive immunity, PKD appears to elicit T_H1_-like activities [46,47]. T_H2_-like responses were also evident as judged by up-regulation of the T_H2_ master transcription factor, GATA3, whereas the trout T_H2_-like cytokine, IL4/13A was refractory towards clinical PKD [48,49]. Other trout IL4/13 isoforms yet to be discovered, however, may play a more prominent role in clinical PKD. The T_H17_ master transcription factor RORγ (a splice variant of RORγ) has not been described in fish to date, although isoforms of RORγ have been sequenced and characterized in rainbow trout [50]. The expression of a trout RORγ transcript did not correlate with either infection read-out. As with IL-4/13A, the existence of more PKD-responsive gene paralogues or other splice variants may also account for the apparent refractoriness of RORγ and the modest changes of trout IL-2 [47,51]. The T_reg_ master transcription factor, FOXP3 exists as two isoforms in trout both of which are up-regulated by the T cell mitogen, Phytohaemagglutinin (PHA) [48]. Here, both isoforms were transcriptionally up-regulated during clinical PKD, which may suggest a role of trout T_reg_-like cells in this disease, particularly as the cytokine mediators of FOXP3 expression, IL-10 and TGF-β1a, were also up-regulated. Evidence for T_H17_-like activities in fish has been provided in the sequencing and characterization of four IL-17 genes, all of which have been shown to respond to bacterial infection and other immune insults, with trout IL17A/F2a demonstrating pro-inflammatory activity [8,52]. As with IL-17, IL-21 and IL-22 are primarily derived from T_H17_ cells and are integral to the functioning and proliferation of the T_H17_ phenotype [53,54]. Initial differentiation of T_H17_ cells is driven by IL-6 and TGF-β and subsequently by IL-21 and TGF-β [19]. Functional studies have confirmed trout IL-21 as a key regulator of B and T cell-like activities inducing IFN-γ, IL-10 and IL-22, and maintaining CD8 and IgM transcript levels in head kidney leucocyte cultures and increasing CD4, T-bet and GATA3 transcripts in the same cells [27]. Here, clinical PKD was associated with marked transcriptional up-regulation of IL-21, IL17A/F2a, and to a lesser extent, IL-22 and IL-17C-1, although only IL-21 correlated positively with parasite prevalence and kidney swelling. These data, in conjunction with up-regulated IL-6 and TGF-β1a suggests that the lymphocytic character of PKD could involve a T_H17_-like activity.

In conclusion, our results imply that PKD pathogenesis is shaped by a profound B cell/antibody response and potentially dysregulated T_H_ cell activity. Our data is also suggestive of phagocyte-mediated pro-inflammatory processes being over-shadowed by a prevailing anti-inflammatory phenotype during PKD pathogenesis, although T_H1_-like mechanisms do appear to be stimulated by PKD. Although many T cell marker and response gene homologues have been identified in fish, there is still a current knowledge gap in terms of the functional characterization of fish T cells. Specific antibodies to immune cell markers and neutralizing antibodies are required to aid future functional assessment of the relative contribution of immune cell populations and immune regulatory molecules to the pathogenesis of PKD and other fish diseases. Such tools will greatly facilitate the identification of immune therapies to drive effector responses towards host protection rather than chronic immunopathogenesis.

## Competing interests

The authors declare that they have no competing interests.

## Authors’ contributions

JWH undertook the sampling and pathological assessment of all fish groups. All authors designed the gene expression study with TW and JWH providing rainbow trout immune gene and parasite RPL18 primer sequences respectively. BG carried out the gene expression experiments. JWH took the lead in writing the manuscript. JWH, TW, and CJS oversaw data analysis performed by BG. BG compiled figures / tables. All authors edited, read, and approved the final manuscript.

## Supplementary Material

Additional file 1 **Summary table of primers used for qPCR analysis.** Oligonucleotide sequences used to detect *T.bryosalmonae* and rainbow trout (*Oncorhynchus mykiss*) genes, amplicon size (bp), and GenBank accession numbers. Click here for file

Additional file 2 **Q-PCR expression profiles of all genes screened during this study.** Data is presented as fold change following initial normalization with trout EF-1α at each swelling grade and subsequently expressed relative to expression levels in control fish. Baseline Cq detection threshold (cCq) has been included as a means of assessing the potential biological relevance of *T*. *bryosalmonae*-mediated changes in immune gene expression. Statistical data (*P*-values) representing gene expression (fold change) at each swelling grade and correlation analysis of gene expression relative to *T*. *bryosalmonae* RPL18 and kidney swelling grade are given. Significant differences (2-tailed) are shown in bold. *P < 0.05, **P < 0.01. Click here for file
