# Supplementary material for: Immune gene expression profiling of Proliferative Kidney Disease in rainbow trout Oncorhynchus mykiss reveals a dominance of anti-inflammatory, antibody and T helper cell-like activities
Source: Vet Res. 2013 Jul 16;44(1):55. doi: 10.1186/1297-9716-44-55 (PMC3733943; doi:10.1186/1297-9716-44-55)
Supplement: Additional file 1 — Summary table of primers used for qPCR analysis. Oligonucleotide sequences used to detect T.bryosalmonae and rainbow trout (Oncorhynchus mykiss) genes, amplicon size (bp), and GenBank accession numbers. [file 1297-9716-44-55-S1.docx]

| **Gene** | **GenBank accession number** | **Forward primer (5’ to 3’)** | **Reverse primer (5’ to 3’)** | **Amplicon size (bp)** |
| --- | --- | --- | --- | --- |
| ***T. bryosalmonae* 18S rDNA** | U70623 | GGACACTGCATGTGCTGCATAGT | CCATGCTAGAATGTCCAGGCACT | 215 |
| ***T. bryosalmonae* RPL18** | FR852769 | GTAAACGGGGACAAAAAGA | GGAGCAGCACCAAAATAC | 251 |
| **EF-1α** | AF498320 | CAAGGATATCCGTCGTGGCA | ACAGCGAAACGACCAAGAGG | 327 |
| **MCSF** | AM901600 | ACCCCGTCTGCCACGAATGA | CAGCTTGGCCCCAGCAACAG | 195 |
| **IL-1β-1** | AJ278242 | CCTGGAGCATCATGGCGTG | GCTGGAGAGTGCTGTGGAAGAACATATAG | 179 |
| **IL-1β-3** | AM181685 | CTGAAGGCCGTCACAATCCA | CTGGTCCTTACAGCGCTCCAA | 195 |
| **COX-2A** | AJ238307 | CCAGTACCAGAACCGTATCGCAG | GTCCACCAGCCACCCTTCC | 200 |
| **COX-2B** | EF175381 | CCAGTATCAGAACCGCATCTCGT | GACCTCCAGCAACCCGTCC | 200 |
| **IL-6** | DQ866150 | CCTTGCGGAACCAACAGTTTG | CCTCAGCAACCTTCATCTGGTC | 288 |
| **IL-11** | AJ867256 | GCTGCTCTCGCTGCTATTGG | AGAGTGGGTCTCATCTCAAGGGA | 250 |
| **TNF-α1** | AJ277604 | TGTGTGGGGTCCTCTTAATAGCAGGTC | CCTCAATTTCATCCTGCATCGTTGA | 102 |
| **TNF-α2** | AJ401377 | CTGTGTGGCGTTCTCTTAATAGCAGCTT | CATTCCGTCCTGCATCGTTGC | 98 |
| **iNOS** | AJ295231 | GACACCTGCCGAGATGATGA | TCCTGAGAGTCCTTTGCCAA | 194 |
| **IL-18** | AJ556990 | GAGCAATGCAAAGCAGATGATTG | CATGTTTTGAGCAGCCAATGTAGTC | 211 |
| **Cathelicidin-1** | AY594646 | ACCAGCTCCAAGTCAAGACTTTGAA | TGTCCGAATCTTCTGCTGCAA | 275 |
| **Cathelicidin-2** | AY542963 | ACATGGAGGCAGAAGTTCAGAAGA | GAGCCAAACCCAGGACGAGA | 135 |
| **Hepcidin-1** | CA369786 | GCTGTTCCTTTCTCCGAGGTGC | GTGACAGCAGTTGCAGCACCA | 165 |
| **LEAP-2A** | AY362186 | GGTTCCTGGTGTTTCTGGTGCT | AGTGGCCACCCCTGCAAAT | 222 |
| **Arginase-1** | BK001403 | CATGTCCTACCTCATCCACGAGC | GATGGGCTTCTTCACCTTTGAGAA | 250 |
| **MCSF-R1** | AJ417832 | GTGAAGGGCAATGCGCGTC | CTGCTGGGACGGGAGAGTAGAAC | 363 |
| **MCSF-R2** | AB091826 | GGTGAAAGGAAATGCCCGTC | GGCCACTCTGCTCTCCTGACTC | 379 |
| **MCSF-1** | AM901600 | AAGACTGAGCCAAACCATCCTAGGAC | GGATAAGGGCTTGGAGTCTCTTCTTCTC | 267 |
| **MCSF-2** | AM949840 | CCTCCCTACAGCACTCTCTCTGACTAC | GGTCAGTACTGTAGGACATCTTGTGTGT | 203 |
| **CD4** | AY973028 | GTGTGGAGGTGCTACAGGTTTTTTC | ATCGTCACCCGCTGTCTGTG | 396 |
| **CD8α** | AF178053 | CCAAGTCGTGCAAAGTGGGAA | CTTGGCTGTCTTTTGTAATGATGTGG | 172 |
| **CD8β** | AY563420 | GAACTATCAAACCCCAGAAGGCTGTG | GACACTTTTTGGGTAGTCGGCTGAA | 125 |
| **CD9** | AF425839 | GTGGCTGTTTTCGGAGACTATG | TGGAACATGGACAGGGTAACAG | 227 |
| **CD83** | AY263797 | GGTGAGGTGGTACAAGCTGG | AAGGGGTGCATCTAGAAGTGG | 427 |
| **IL-2Rβ** | FN813346 | CGGTGGAAACTTTCAGAAATGGCT | AAGAGGCTGCTGGGGTATTTGGT | 261 |
| **IgT H-secretory** | AY870263 | CATCAGCTTCACCAAAGGAAGTGA | TCACTTGTCTTCACATGAGTTACCCGT | 361 |
| **IgT H-membrane** | AY870265 | TCGAAGTCCACGGCGAACA | GTGTTCTTCACCGCTTCATCTTGAA | 187 |
| **IgM H-secretory** | X65261 | TACAAGAGGGAGACCGGAGGAGT | CTTCCTGATTGAATCTGGCTAGTGGT | 221 |
| **IgM H-membrane** | OMU04616 | CCTACAAGAGGGAGACCGATTGTC | GTCTTCATTTCACCTTGATGGCAGT | 168 |
| **IgD H-secretory** | AY870262 | TGAACATATCCAAACCAGGTGTCTG | GTCCTGAAGTCATCATTTTGTCTTGA | 357 |
| **IgD H-membrane** | AY870261 | TGAACATATCCAAACCAGAGCTCC | GTCCTGAAGTCATCATTTTGTCTTGA | 191 |
| **T-bet** | FM863825 | GGTAACATGCCAGGGAACAGGA | TGGTCTATTTTTAGCTGGGTGATGTCTG | 317 |
| **IL-2** | AM422779 | CATGTCCAGATTCAGTCTTCTATACACC | GAAGTGTCCGTTGTGCTGTTCTC | 211 |
| **IFNγ** | AJ616215 | CAAACTGAAAGTCCACTATAAGATCTCCA | TCCTGAATTTTCCCCTTGACATATTT | 210 |
| **Type I IFN-A** | AJ580911 | CTGTTTGATGGGAATATGAAATCTGC | CCTGTGCACTGTAGTTCATTTTTCTCAG | 193 |
| **GATA3** | FM863826 | CCAAAAACAAGGTCATGTTCAGAAGG | TGGTGAGAGGTCGGTTGATATTGTG | 313 |
| **IL-4/13A** | FN820501 | ACCACCACAAAGTGCAAGGAGTTCT | CACCTGGTCTTGGCTCTTCACAAC | 156 |
| **RORγ** | FM883712 | ACAGACCTTCAAAGCTCTTGGTTGTG | GGGAAGCTTGGACACCATCTTTG | 262 |
| **IL-21** | FM883702 | CAACAGTGTGATGTCGAACGCTC | CCTTGGCAGACTGTTTTCTCTCTCC | 210 |
| **IL-22** | AM748538 | GAAGGAACACGGCTGTGCTATTAAAC | GATCTAGGCGTGCACACAGAAGTC | 168 |
| **IL-17A/F2a** | AJ580842 | CGTGTCGAAGTACCTGGTTGTGT | GGTTCTCCACTGTAGTGCTTTTCCA | 212 |
| **IL-17C-1** | FM955455 | CTGGCGGTACAGCATCGATA | GAGTTATATCCATAATCTTCGTATTCGGC | 138 |
| **IL-17C-2** | FM955456 | CTGGCGGTACAGCATCGATA | CAGAGTTATATGCATGATGTTGGGC | 134 |
| **IL-17D** | AJ580843 | GAAGAAATCCTCGAGCAGATGTTTG | GGGTCGTGGGAGATCCTGTATG | 200 |
| **FOXP3A** | FM883710 | CCCAGAACCGAGGTGGAGTGT | TGACGGACAGCGTTCTTCCA | 319 |
| **FOXP3B** | FM883711 | TCCTGCCCCAGTACTCATCCC | TGACGGACAGCGTTCTTCCA | 295 |
| **TGF-β1a** | AJ007836 | CTCACATTTTACTGATGTCACTTCCTGT | GGACAACTGCTCCACCTTGTG | 371 |
| **IL-10A** | AB118099 | GGATTCTACACCACTTGAAGAGCCC | GTCGTTGTTGTTCTGTGTTCTGTTGT | 119 |
| **IL-10B** | FR691804 | GGGATTCTAGACCACATCAAGAGTCC | GATGGGAGATTTAAAGTTGTGTGTTCC | 132 |
| **nIL-1F** | AJ555869 | CCCATTCCTCGTGACACCAG | CTGGACGACCTGGAGAGTGACT | 250 |
| **CISHa** | AM903340 | CATTCTACCTTGATACCTCAGGCTGGT | ACATCAGGGAAGGACAGAAGGCT | 247 |
| **SOCS-1** | AM748721 | GATTAATACCGCTGGGATTCTGTG | CTCTCCCATCGCTACACAGTTCC | 136 |
| **SOCS-2** | AM748722 | GGATCCACTCGCAAATAGGACGATAC | GGATTCGGGTGAGTGGCAGGT | 184 |
| **SOCS-3** | AM748723 | CACAGAGAAACCGTTAAAAGGACTATCC | AAGGGGCTGCTGCTCATGAC | 228 |
| **SOCS-5b** | AM903341 | ACTAACTAGCTATCTTTTGGAGAGCAGCA | CGGCATCTCTTCTCTCCTCAGACC | 322 |
| **SOCS-7** | AM903343 | GAACTGGAAAAATGTGGCTGGT | GACACCAGAGGCTGAAGGTTCC | 202 |
| **IL-15** | AJ628345 | TATTGAGCTGCCTGAGTGCCAC | AAGATGCAGTGGTCATTGTAGATGTC | 239 |
| **VEGF** | AJ717302 | CGAAAGTGTGAATGCAGACCAA | GTCTAATTGTGTGAATTTGCAGGAACA | 141 |
| **M17** | FM866399 | GTGGACCTCTTAAAAACATACAAGCTCAG | GGATGGTGGCTGTAAGTCTGTCTG | 204 |
| **CNTF** | FM866401 | GCACTTATCTTCTGGAGCTATATAGGGAGA | AACTCCATCAACCTCCTCATTGC | 341 |
