# Supplementary material for: Immune gene expression profiling of Proliferative Kidney Disease in rainbow trout Oncorhynchus mykiss reveals a dominance of anti-inflammatory, antibody and T helper cell-like activities
Source: Vet Res. 2013 Jul 16;44(1):55. doi: 10.1186/1297-9716-44-55 (PMC3733943; doi:10.1186/1297-9716-44-55)
Supplement: Additional file 2 — Q-PCR expression profiles of all genes screened during this study. Data is presented as fold change following initial normalization with trout EF-1α at each swelling grade and subsequently expressed relative to expression levels in control fish. Baseline Cq detection threshold (cCq) has been included as a means of assessing the potential biological relevance of T. bryosalmonae-mediated changes in immune gene expression. Statistical data (P-values) representing gene expression (fold change) at each swelling grade and correlation analysis of gene expression relative to T. bryosalmonae RPL18 and kidney swelling grade are given. Significant differences (2-tailed) are shown in bold. *P < 0.05, **P < 0.01. [file 1297-9716-44-55-S2.docx]

| **Gene** | **Grade 0 average cCq** |  | **Grade 1** | **Grade 1-2** | **Grade 2** | **Grade 3** | **Pearson correlation** | | | |
| --- | --- | --- | --- | --- | --- | --- | --- | --- | --- | --- |
|  |  |  |  |  |  |  | **To *T. bryosalmonae* RPL18** | | **To**  **kidney swelling grade** | |
| ***T.bryosalmonae* 18S rDNA** | *No detectable* | R.E.^1^ | 2105.63 | 4266.94 | 6652.34 | 8034.26 | **.936**** | *r* | | **.755**** |
|  |  | *P*-value | .000 | .000 | .000 | .000 | **.000** | *P-*value | | **.000** |
| ***T.bryosalmonae* RPL18** |  | R.E.^2^ | 8013.47 | 37233.66 | 39385.81 | 26654.46 | *1.000* | *r* | | **.740**** |
|  |  | *P*-value | .000 | .000 | .000 | .000 | + | *P-*value | | .000 |
| **IL-1β-1** | 27.16 | Fold | 3.71 | 2.24 | 2.10 | 2.31 | .277 | *r* | | -.116 |
|  |  | *P*-value | .010 | .110 | .200 | .960 | .076 | *P-*value | | .463 |
| **IL-1β-3** | 23.15 | Fold | 1.80 | 0.73 | 0.94 | 1.06 | -.172 | *r* | | -.189 |
|  |  | *P*-value | .275 | .353 | .553 | .441 | .277 | *P-*value | | .229 |
| **TNF-α1** | 24.52 | Fold | 1.12 | 0.95 | 0.94 | 0.84 | -.108 | *r* | | -.155 |
|  |  | *P*-value | .574 | .693 | .563 | .519 | .509 | *P-*value | | .339 |
| **TNF-α2** | 26.60 | Fold | 0.97 | 2.19 | 1.52 | 1.64 | .305 | *r* | | .106 |
|  |  | *P*-value | .984 | .026 | .557 | .448 | .056 | *P-*value | | .514 |
| **IL-6** | 34.12 | Fold | 7.44 | 31.53 | 23.59 | 15.38 | **.850**** | *r* | | **.510**** |
|  |  | *P*-value | .002 | .000 | .000 | .000 | **.000** | *P-*value | | **.001** |
| **IL-11** | 29.62 | Fold | 31.74 | 25.04 | 14.13 | 11.54 | **.656**** | *r* | | **.399**** |
|  |  | *P*-value | .000 | .000 | .000 | .000 | **.000** | *P-*value | | **.009** |
| **M17** | 26.05 | Fold | 5.10 | 6.28 | 5.48 | 2.60 | **.525**** | *r* | | .179 |
|  |  | *P*-value | .002 | .000 | .001 | .061 | **.000** | *P-*value | | .257 |
| **IL-18** | 22.64 | Fold | 4.08 | 4.12 | 5.15 | 3.51 | **.474**** | *r* | | .255 |
|  |  | *P*-value | .013 | .035 | .018 | .040 | **.002** | *P-*value | | .104 |
| **COX-2A** | 24.69 | Fold | 2.15 | 1.68 | 1.75 | 1.07 | .142 | *r* | | -.161 |
|  |  | *P*-value | .132 | .311 | .371 | .516 | .370 | *P-*value | | .308 |
| **COX-2B** | 32.73 | Fold | 3.59 | 3.94 | 3.10 | 2.03 | **.360*** | *r* | | .164 |
|  |  | *P*-value | .141 | .011 | .021 | .223 | **.019** | *P-*value | | .299 |
| **MCSF-1** | 22.92 | Fold | 1.18 | 0.77 | 0.49 | 0.23 | **-.433**** | *r* | | **-.658**** |
|  |  | *P*-value | .936 | .505 | .023 | .000 | **.004** | *P-*value | | **.000** |
| **MCSF-2** | 21.78 | Fold | 1.81 | 3.82 | 2.77 | 1.41 | .157 | *r* | | -.076 |
|  |  | *P*-value | .227 | .048 | .240 | .864 | .322 | *P-*value | | .635 |
| **MCSF-R1** | 27.65 | Fold | 1.05 | 2.48 | 1.01 | 0.57 | -.060 | *r* | | **-.375*** |
|  |  | *P*-value | .997 | .196 | .567 | .023 | .707 | *P-*value | | **.014** |
| **MCSF-R2** | 21.77 | Fold | 2.15 | 2.70 | 2.65 | 1.22 | .234 | *r* | | -.001 |
|  |  | *P*-value | .115 | .126 | .160 | .748 | .135 | *P-*value | | .995 |
| **iNOS** | 33.31 | Fold | 2.34 | 5.54 | 2.43 | 1.28 | **.362*** | *r* | | -.035 |
|  |  | *P*-value | .177 | .034 | .338 | .834 | **.019** | *P-*value | | .825 |
| **Arginase-1** | 15.20 | Fold | 1.05 | 0.50 | 0.45 | 0.23 | **-.496**** | *r* | | **-.612**** |
|  |  | *P*-value | .688 | .047 | .047 | .000 | **.001** | *P-*value | | **.000** |
| **Cathelicidin-1** | 28.63 | Fold | 52.60 | 136.21 | 72.80 | 57.81 | **.807**** | *r* | | **.530**** |
|  |  | *P*-value | .000 | .000 | .000 | .000 | **.000** | *P-*value | | **.000** |
| **Cathelicidin-2** | 29.58 | Fold | 9.00 | 56.40 | 94.90 | 19.10 | **.814**** | *r* | | **.547**** |
|  |  | *P*-value | .001 | .000 | .000 | .000 | **.000** | *P-*value | | **.000** |
| **Hepcidin-1** | 25.76 | Fold | 5.36 | 10.85 | 22.37 | 6.42 | **.723**** | *r* | | **.360*** |
|  |  | *P*-value | .012 | .001 | .000 | .008 | **.000** | *P-*value | | **.019** |
| **LEAP-2A** | 34.31 | Fold | 10.92 | 12.87 | 7.76 | 6.84 | **.641**** | *r* | | .281 |
|  |  | *P*-value | .001 | .000 | .001 | .007 | **.000** | *P-*value | | .071 |
| **CD8α** | 25.31 | Fold | 5.37 | 4.01 | 5.46 | 3.91 | **.422**** | *r* | | .238 |
|  |  | *P*-value | .001 | .006 | .001 | .029 | **.005** | *P-*value | | .129 |
| **CD8β** | 21.41 | Fold | 2.99 | 3.05 | 3.34 | 2.33 | **.341*** | *r* | | .128 |
|  |  | *P*-value | .015 | .029 | .013 | .214 | **.027** | *P-*value | | .419 |
| **IL-2Rβ** | 24.63 | Fold | 3.79 | 5.19 | 1.28 | 0.93 | .214 | *r* | | -.168 |
|  |  | *P*-value | .002 | .003 | .272 | .913 | .174 | *P-*value | | .289 |
| **IgD H-secretory** | 21.39 | Fold | 0.98 | 1.02 | 0.89 | 0.92 | -.104 | *r* | | -.095 |
|  |  | *P*-value | .783 | .745 | .492 | .630 | .521 | *P-*value | | .561 |
| **IgD H-membrane** | 19.34 | Fold | 0.94 | 1.15 | 1.11 | 1.29 | .199 | *r* | | .293 |
|  |  | *P*-value | .850 | .498 | .544 | .101 | .217 | *P-*value | | .067 |
| **IgM H-secretory** | 14.14 | Fold | 5.39 | 3.38 | 8.80 | 4.80 | **.554**** | *r* | | **.399**** |
|  |  | *P*-value | .011 | .062 | .001 | .005 | **.000** | *P-*value | | **.009** |
| **IgM H-membrane** | 17.38 | Fold | 1.73 | 1.19 | 1.02 | 0.64 | -.020 | *r* | | -.270 |
|  |  | *P*-value | .187 | .668 | .993 | .219 | .899 | *P-*value | | .084 |
| **IgT H-secretory** | 21.31 | Fold | 8.19 | 24.68 | 51.03 | 68.62 | **.747**** | *r* | | **.732**** |
|  |  | *P*-value | .028 | .000 | .000 | .000 | **.000** | *P-*value | | **.000** |
| **IgT H-membrane** | 21.06 | Fold | 1.00 | 2.32 | 2.21 | 2.96 | **.630**** | *r* | | **.581**** |
|  |  | *P*-value | .345 | .001 | .002 | .000 | **.000** | *P-*value | | **.000** |
| R.E.^1^ = Relative expression to MCSF | | | |  | ***** Correlation is significant at the .05 level (2-tailed) | | | | | |
| R.E.^2^ = Relative expression to EF-1α | | | |  | ****** Correlation is significant at the .01 level (2-tailed) | | | | | |

| **Gene** | **Grade 0 average cCq** |  | **Grade 1** | **Grade 1-2** | **Grade 2** | **Grade 3** | **Pearson correlation** | | | |
| --- | --- | --- | --- | --- | --- | --- | --- | --- | --- | --- |
|  |  |  |  |  |  |  | **To *T. bryosalmonae* RPL18** | | **To**  **kidney swelling grade** | |
| **CD4** | 22.47 | Fold | 3.66 | 4.28 | 4.72 | 3.56 | **.517**** | *r* | | .298 |
|  |  | *P*-value | .009 | .005 | .002 | .020 | **.000** | *P-*value | | .055 |
| **CD9** | 15.95 | Fold | 1.58 | 0.80 | 2.02 | 0.91 | .013 | *r* | | -.111 |
|  |  | *P*-value | .349 | .952 | .423 | .560 | .937 | *P-*value | | .485 |
| **CD83** | 20.86 | Fold | 1.88 | 1.53 | 1.72 | 1.12 | .185 | *r* | | -.105 |
|  |  | *P*-value | .104 | .324 | .416 | .830 | .242 | *P-*value | | .507 |
| **T-bet** | 24.94 | Fold | 7.00 | 6.86 | 7.06 | 2.80 | **.548**** | *r* | | .203 |
|  |  | *P*-value | .000 | .000 | .000 | .008 | **.000** | *P-*value | | .197 |
| **IL-2** | 28.68 | Fold | 3.68 | 4.16 | 2.16 | 2.54 | **.454**** | *r* | | .048 |
|  |  | *P*-value | .010 | .006 | .151 | .258 | **.003** | *P-*value | | .761 |
| **IFNγ** | 29.25 | Fold | 5.06 | 15.40 | 10.03 | 8.49 | **.716**** | *r* | | **.500**** |
|  |  | *P*-value | .007 | .000 | .000 | .000 | **.000** | *P-*value | | **.001** |
| **Type I IFN-A** | 31.73 | Fold | 4.68 | 3.46 | 2.95 | 1.73 | **.375*** | *r* | | -.032 |
|  |  | *P*-value | .001 | .002 | .042 | .443 | **.014** | *P-*value | | .843 |
| **GATA3** | 25.00 | Fold | 4.64 | 5.77 | 3.63 | 2.33 | **.490**** | *r* | | .115 |
|  |  | *P*-value | .001 | .000 | .012 | .069 | **.001** | *P-*value | | .470 |
| **IL-4/13A** | 25.77 | Fold | 1.67 | 2.71 | 1.70 | 0.80 | .193 | *r* | | -.124 |
|  |  | *P*-value | .206 | .206 | .558 | .715 | .222 | *P-*value | | .433 |
| **RORγ** | 25.69 | Fold | 2.44 | 1.57 | 1.33 | 0.71 | -.055 | *r* | | **-.323*** |
|  |  | *P*-value | .101 | .283 | .812 | .110 | .728 | *P-*value | | **.037** |
| **IL-21** | 33.78 | Fold | 20.28 | 42.62 | 31.58 | 11.91 | **.737**** | *r* | | **.506**** |
|  |  | *P*-value | .000 | .000 | .000 | .000 | **.000** | *P-*value | | **.001** |
| **IL-22** | 33.65 | Fold | 10.03 | 6.67 | 5.49 | 4.48 | **.420**** | *r* | | .159 |
|  |  | *P*-value | .012 | .005 | .014 | .119 | **.006** | *P-*value | | .315 |
| **IL-17A/F2a** | 28.27 | Fold | 10.84 | 11.23 | 2.64 | 3.24 | **.432**** | *r* | | .061 |
|  |  | *P*-value | .001 | .001 | .106 | .108 | **.004** | *P-*value | | .700 |
| **IL-17C-1** | 27.92 | Fold | 6.56 | 2.74 | 1.43 | 0.84 | .084 | *r* | | -.259 |
|  |  | *P*-value | .001 | .031 | .676 | .489 | .599 | *P-*value | | .097 |
| **IL-17C-2** | 30.90 | Fold | 2.08 | 1.36 | 1.06 | 0.70 | .018 | *r* | | **-.349*** |
|  |  | *P*-value | .115 | .500 | .911 | .085 | .911 | *P-*value | | **.024** |
| **IL-17D** | 31.72 | Fold | 4.95 | 5.73 | 3.78 | 3.56 | **.478**** | *r* | | .259 |
|  |  | *P*-value | .002 | .000 | .002 | .013 | **.001** | *P-*value | | .097 |
| **FOXP3A** | 26.19 | Fold | 2.96 | 5.15 | 4.75 | 2.52 | **.374*** | *r* | | .042 |
|  |  | *P*-value | .055 | .032 | .095 | .475 | **.015** | *P-*value | | .792 |
| **FOXP3B** | 27.03 | Fold | 4.02 | 3.87 | 3.13 | 2.11 | **.444**** | *r* | | .109 |
|  |  | *P*-value | .001 | .000 | .002 | .130 | **.003** | *P-*value | | .493 |
| **TGF-β1a** | 22.87 | Fold | 3.34 | 3.00 | 2.02 | 1.59 | **.358*** | *r* | | -.015 |
|  |  | *P*-value | .010 | .008 | .114 | .506 | **.020** | *P-*value | | .926 |
| **IL-10A** | 26.26 | Fold | 14.68 | 18.21 | 25.24 | 9.51 | **.735**** | *r* | | **.414**** |
|  |  | *P*-value | .001 | .000 | .000 | .001 | **.000** | *P-*value | | **.006** |
| **IL-10B** | 29.50 | Fold | 7.51 | 8.03 | 12.73 | 5.73 | **.679**** | *r* | | **.375*** |
|  |  | *P*-value | .000 | .000 | .000 | .001 | **.000** | *P-*value | | **.014** |
| **nIL-1F** | 24.76 | Fold | 3.14 | 3.32 | 2.81 | 1.97 | **.443**** | *r* | | .136 |
|  |  | *P*-value | .014 | .008 | .020 | .163 | **.003** | *P-*value | | .392 |
| **CISHa** | 21.78 | Fold | 2.29 | 2.11 | 3.78 | 1.36 | .299 | *r* | | .064 |
|  |  | *P*-value | .144 | .190 | .019 | .675 | .054 | *P-*value | | .687 |
| **SOCS-1** | 21.03 | Fold | 3.35 | 4.47 | 7.08 | 3.87 | **.613**** | *r* | | **.396**** |
|  |  | *P*-value | .011 | .002 | .000 | .004 | **.000** | *P-*value | | **.009** |
| **SOCS-2** | 22.56 | Fold | 1.61 | 0.84 | 0.85 | 0.35 | -.235 | *r* | | **-.502**** |
|  |  | *P*-value | .690 | .408 | .308 | .002 | .133 | *P-*value | | **.001** |
| **SOCS-3** | 22.74 | Fold | 3.44 | 8.38 | 10.24 | 5.90 | **.770**** | *r* | | **.472**** |
|  |  | *P*-value | .016 | .000 | .000 | .001 | **.000** | *P-*value | | **.002** |
| **SOCS-5b** | 27.37 | Fold | 2.33 | 1.79 | 1.31 | 0.97 | .255 | *r* | | -.112 |
|  |  | *P*-value | .008 | .046 | .193 | .972 | .103 | *P-*value | | .478 |
| **SOCS-7** | 28.17 | Fold | 1.74 | 1.43 | 1.13 | 0.90 | .144 | *r* | | -.185 |
|  |  | *P*-value | .065 | .228 | .534 | .544 | .364 | *P-*value | | .240 |
| **IL-15** | 21.25 | Fold | 7.88 | 3.12 | 3.88 | 1.90 | **.469**** | *r* | | .138 |
|  |  | *P*-value | .000 | .002 | .000 | .047 | **.002** | *P-*value | | .383 |
| **VEGF** | 27.74 | Fold | 2.19 | 3.64 | 2.63 | 0.92 | .147 | *r* | | -.178 |
|  |  | *P*-value | .187 | .040 | .228 | .392 | .351 | *P-*value | | .259 |
| **CNTF** | 25.64 | Fold | 1.40 | 0.88 | 1.24 | 0.79 | -.058 | *r* | | -.244 |
|  |  | *P*-value | .326 | .930 | .777 | .261 | .713 | *P-*value | | .119 |
| * Correlation is significant at the .05 level (2-tailed) | | | | | | | | | | |
| ** Correlation is significant at the .01 level (2-tailed) | | | | | | | | | | |
